# A transcript profiling approach reveals the zinc finger transcription factor ZNF191 is a pleiotropic factor

**DOI:** 10.1186/1471-2164-10-241

**Published:** 2009-05-22

**Authors:** Jianzhong Li, Xia Chen, Xuelian Gong, Ying Liu, Hao Feng, Lei Qiu, Zhenlin Hu, Junping Zhang

**Affiliations:** 1 Department of Biochemical Pharmacy, Second Military Medical University, Shanghai, PR China; 2 Shanghai Research Center of Biotechnology, Chinese Academy of Sciences, Shanghai, PR China

## Abstract

**Background:**

The human zinc finger protein 191 (ZNF191) is a member of the SCAN domain family of Krüppel-like zinc finger transcription factors. ZNF191 shows 94% identity to its mouse homologue zinc finger protein 191(Zfp191), which is the most highly conserved among the human-mouse SCAN family member orthologues pairs. Zfp191 is widely expressed during early embryogenesis and in adult organs. Moreover, Zfp191^-/- ^embryos have been shown to be severely retarded in development and die approximately at embryonic day E7.5. ZNF191 can specifically interact with the widespread TCAT motif which constitutes the HUMTH01 microsatellite in the tyrosine hydroxylase (TH) gene. Allelic variations of HUMTH01 have been stated to have a quantitative silencing effect on TH gene expression and to correlate with quantitative and qualitative changes in the binding by ZNF191. In addition, ZNF191 displays a suppressive effect on the transcription; however, little downstream targets have identified.

**Results:**

We searched for ZNF191 target genes by using a transient overexpression and knockdown strategy in the human embryo kidney (HEK293) cells. Microarray analyses identified 6094 genes modulated by overexpression of *ZN*F191 and 3332 genes regulated by knockdown of ZNF191, using a threshold of 1.2-fold. Several interested candidate genes, validated by real time RT-PCR, were correlated well with the array data. Interestingly, 1456 genes were identified in both transient overexpression and transient knockdown strategies. The GenMAPP and MappFinder software packages were further used for pathway analysis of these significantly altered genes. Several gene pathways were found to be involved in processes of the regulation of kinase activity, transcription, angiogenesis, brain development and response to DNA damage.

**Conclusion:**

Our analysis reveals for the first time that ZNF191 is a pleiotropic factor that has a role in hematopoiesis, brain development and cancers.

## Background

The regulation of gene expression in response to intrinsic and extrinsic cues is a fundamental cellular process in the growth and development of organisms. Critical to the control of gene expression are transcription factors that bind to specific DNA sequences and subsequently modulate gene transcription [1]. A significant number of transcription factors use a conserved zinc finger domain to bind their target DNAs. Zinc finger factors participate in a variety of cellular activities, such as development and differentiation, and play a role in human disease. In fact, the human genome encompasses approximately 600 to700 genes that contain a particular C_2_H_2_-type of zinc finger, which employs two cysteine and two histidine amino acid residues to coordinate the single zinc atom in the finger-like structure [2-4]. In the C_2_H_2_-type of zinc finger proteins, there is a highly conserved consensus sequence TGEKP(F/Y)X (X representing any amino acid) between adjacent zinc finger motifs. The zinc finger proteins containing this specific structure are termed Krüppel-like zinc finger proteins because the structure was first found in *Drosophila *Krüppel protein [5,6]. Many Krüppel-like factors exhibit diverse regulatory functions in cell growth, proliferation, differentiation, and embryogenesis [7-10]. The SCAN (SRE-ZBP, Ctfin 51, AW-1, and Number 18) [11,12], which is also known as the leucine rich region (LeR) [12], KRAB (Krüppel-associated box) [10,13], and POZ (poxvirus and zinc finger) [14] domains are highly conserved modules found in the N-terminus of C_2_H_2 _zinc finger transcription factors that have been demonstrated to mediate specific protein-protein interactions [15-18]. The SCAN domain is a conserved motif that appears to control the association of SCAN-containing proteins into non-covalent complexes, and may be the primary mechanism underlying partner choice in the dimerization of these transcription factors [16,19-22]. The genes encoding SCAN domains are clustered, often in tandem arrays, in both the human and mouse genomes and are capable of generating isoforms that may affect the function of family members. Although the function of most of the family members is not known, some of the SCAN domain family members play roles in cell survival and differentiation or development [16,23-25]. Like the KRAB motif, the SCAN domain-containing C_2_H_2 _zinc finger proteins are unique to the vertebrate lineage [2,3].

Human zinc finger protein 191 (ZNF191, also known as ZNF24 and KOX(17) [10]) is a member of the SCAN domain family of Krüppel-like zinc finger transcription factors [15]. This gene is initially named as *RSG-A *(for retinoic acid suppressed gene-A) because its mRNA can be amplified by homologous RT-PCR only in retinoic acid-untreated but not in retinoic acid-treated acute promyelocytic leukemia NB4 cells[26]. ZNF191 shows 94% identity to its mouse homologue zinc finger protein 191(Zfp191, also called ZF-12), which is the most highly conserved among the human-mouse SCAN family member orthologues pairs[16]. Tissue mRNA analysis showed that *ZNF191 *gene was ubiquitously expressed [26,27]. The mouse *Zfp191 *has been previously isolated from chondrocytic and mesenchymal precursor cell lines using a subtractive hybridization screening. *Zfp191 *mRNA is expressed during embryonic development and in different organs in adult, including rib cartilage, suggesting that *Zfp191 *may have a role in cartilage differentiation and in basic cellular processes[28].

A study demonstrated that ZNF191 **c**an specifically bind to the TCAT repeats (HUMTH01) in the first intron of the human tyrosine hydroxylase (*TH*) gene, which encodes the rate-limiting enzyme in the synthesis of catecholamines [27,29]. Allelic variations of HUMTH01 have a quantitative silencing effect on *TH *gene expression in vitro, and correlate with quantitative and qualitative changes in the binding by ZNF191 [27]. *ZNF191 *mRNA levels was present at high levels in catecholaminergic tissues including the substantia nigra, the hypothalamus and the olfactory bulb. In the rat embryo, the mRNA was present at high levels in total embryonic head, which may reflect a role in brain development [27]; however, the molecular mechanisms remain unknown.

The *ZNF191 *gene is located on chromosome 18q12.1[10,26], a region frequently deleted in colorectal carcinomas, suggesting a possible role in the negative regulation of tumor growth[30,31]. ZNF191 contains four continuous typical C_2_H_2 _zinc fingers in its C-terminus, and one SCAN domain in its N-terminus [10,26]. Biochemical binding study shows the SCAN domain of ZNF191 as a selective oligomerization domain[20]. The SCAN domain of ZNF191 displays a suppressive effect on the transcription in CHO and NIH3T3 cells[26]; however, little else is known regarding its role in gene expression.

In the present study, we transiently overexpressed and silenced *ZNF191 *gene in the human embryo kidney (HEK293) cells and then applied microarray assay to identify possible *ZNF191 *target genes. Expression of interested candidate genes was also examined by real time RT-PCR, and these data correlated well with the array data. In general, our data reveal that ZNF191 modulates the expression of a large number of genes that are implicated in regulation of kinase activity, regulation of transcription, angiogenesis, brain development and response to DNA damage. Our analysis reveals for the first time that ZNF191 is a pleiotropic factor that has a role in hematopoiesis, brain development, tumor growth.

## Results

### Data analysis: general features

**Figure 1:**
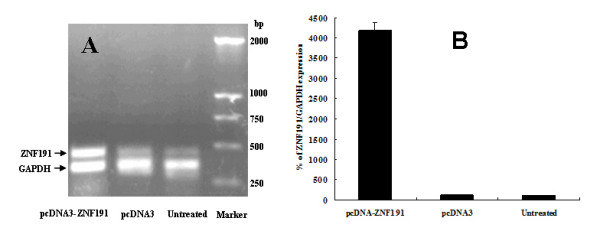
**RT-PCR analysis of the *ZNF191 *gene expression in HEK293 cells transfected with pcDNA3-ZNF191 or pcDNA3 and untreated cells**. (A) Semiquantitative RT-PCR analysis of the *ZNF191 *gene expression in the transfected cells and untreated cells. Total cellular RNA was isolated from pcDNA3-ZNF191 or pcDNA3 transfectants and untreated cells, and, after reverse transcription, PCR was performed with ZNF191- and GAPDH- specific primers. The up-regulation of the 481-bp ZNF191-specific band was detected in cells transfected with pcDNA3-ZNF191 plasmid. (B) Quantitative RT-PCR analysis of the relative ZNF191/GAPDH expression in the transfected cells and untreated cells.

**Figure 2:**
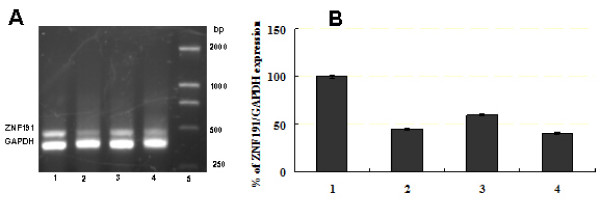
**RT-PCR analysis of the *ZNF191 *gene expression in HEK293 cells transfected with *ZNF191 *siRNA expression vectors or empty plasmid**. (A) Semiquantitative RT-PCR analysis of the *ZNF191 *gene expression in the transfected cells. RT-PCR and gel electrophoresis of amplified products. The sizes of the amplified products are 481 bp (ZNF191) and 381 bp (GAPDH). Lane 1, the control cells (transfected with empty vector). Lane 2, lane 3, or lane 4, HEK293 cells transfected with pSZNF191-1, -2, or -3 (ZNF191 siRNA expression vectors), respectively. Lane 5, DNA size marker. (B) Quantitative RT-PCR analysis of the relative *ZNF191*/*GAPDH *expression in the transfected cells. Lane 1, the control cells (transfected with empty vector). Lane 2, lane 3, or lane 4, HEK293 cells transfected with pSZNF191-1, -2, or -3, respectively.

To identify ZNF191 target genes, a combined approach of transient overexpression and transient knockdown (KD) are used to identify genes modified by the ZNF191 transcription factor with 44 K Whole-Genome 60-mer oligonucleotide microarrays, using a cellular model (HEK293). Plasmids overexpressing *ZNF191 *cDNA (pcDNA3-ZNF191) or control empty pcDNA3 vectors were transiently transfected into HEK293 cells for 24 hours. As expected, introduction of pcDNA3-ZNF191 in HEK293 cells resulted in higher expression of *ZNF191 *mRNA than in cells transfected with control empty plasmid or untreated cells (Figure 1A). Real-time PCR results showed that the *ZNF191 *expression level was increased > 30 times in cells transfected with pcDNA3-ZNF191 compared with control cells (Figure 1B). For the reduction of *ZNF191 *expression, three hairpin siRNA expression vectors against *ZNF191 *were generated [32] and the knock-down of ZNF191 was assessed 48 hours after transfection of the HEK293 cells. Figure 2 showed that the three siRNA expression vectors were found to efficiently eliminate the target mRNA. The reduction of ZNF191 mRNA levels in transfected cells appears to be approximately 60% at the best compared to the control level. Due to no greater inhibition of *ZNF191 *mRNA at longer times than 48 hours, transfection of the HEK293 cells with the most efficient vector for 48 h was used in subsequent experiments.

RNA samples were prepared from the transient overexpression and transient knockdown cells as well as control cells. Probe labelling, hybridization, and scanning were done by Shanghai Biochip Co., Ltd (China). The complete list of genes with differential expression can be found at the website . Microarray showed that 3273 genes were up-regulated more than 1.2-fold, while 3631 were down-regulated by the overexpression of the *ZNF191 *gene (see Additional file 1), while 2111 were up-regulated by the KD and 1221 were down-regulated, using the same threshold (see Additional file 2). Interestingly, 1456 genes were identified in both situations, and 1344 (92%) behaved consistently between the two experiments (i.e. induced by the overexpression and repressed by the KD, or the other way around) (see Additional file 3). These identified genes were subsequently used in pathway analysis with MappFinder.

Using a threshold of 2 for data analysis, 592 genes were found to be regulated by the overexpression with 143 genes up-regulated and 449 down-regulated (see Additional file 1, highlighted data) while 172 genes were regulated by the KD with 83 genes up-regulated and 89 down-regulated (see Additional file 2, highlighted data). The overlap was found between transient overexpression and transient knockdown with 69 shared genes(see Additional file 3, highlighted data). Thus, ZNF191 appears to modulate multiple transcriptional events, acting both as an activator and a repressor.

### Pathway analysis of the ZNF191 transient overexpression and knockdown signature

To further evaluate our microarray data, GenMAPP and MAPPFinder software  were used to organize gene expression data into MAPPs (microarray pathway profiles) that represent specific biological pathways and functionally grouped genes, based on the GO system. Three main classes of branches in the GO tree are biological processes, molecular functions and cellular components [33].

**Figure 3:**
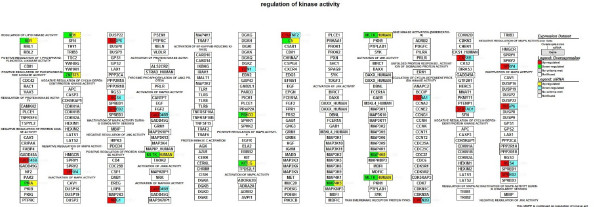
**Gene list involved in the regulation of kinase activity pathway generated by GenMAPP**. The color on the left side of gene box illustrates the gene changes by *ZNF191 *overexpression; the color on the right indicates the gene changes by siRNA-mediated knockdown of *ZNF191*. Red and green indicates up-regulated and down-regulated in *ZNF191 *overexpression experiment, respectively. Yellow and blue indicates up-regulated and down-regulated in siRNA experiment, respectively. Grey indicates that selection criteria were not met but the gene was represented in the array. White boxes indicate that the gene was not present in the chip.

**Figure 4:**
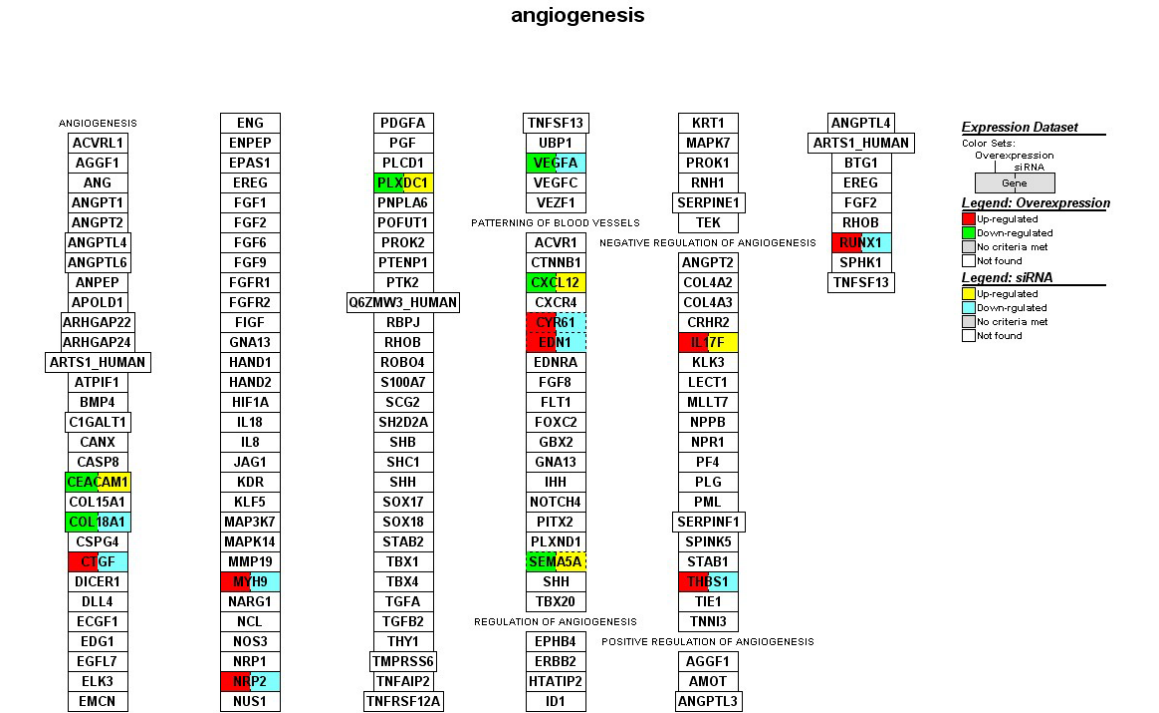
**Gene list involved in the angiogenesis pathway generated by GenMAPP**. The color on the left side of gene box illustrates the gene changes by *ZNF191 *overexpression; the color on the right indicates the gene changes by siRNA-mediated knockdown of *ZNF191*. Red and green indicates up-regulated and down-regulated in *ZNF191 *overexpression experiment, respectively. Yellow and blue indicates up-regulated and down-regulated in siRNA experiment, respectively. Grey indicates that selection criteria were not met but the gene was represented in the array. White boxes indicate that the gene was not present in the chip.

**Figure 5:**
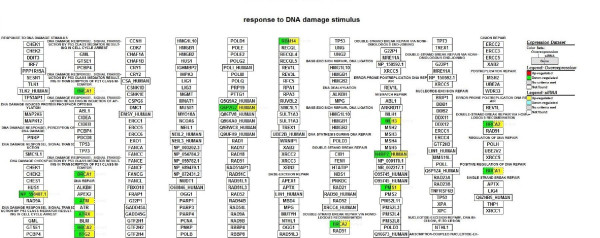
**Gene list involved in the response to DNA damage stimulus pathway generated by GenMAPP**. The color on the left side of gene box illustrates the gene changes by *ZNF191 *overexpression; the color on the right indicates the gene changes by siRNA-mediated knockdown of *ZNF191*. Red and green indicates up-regulated and down-regulated in *ZNF191 *overexpression experiment, respectively. Yellow and blue indicates up-regulated and down-regulated in siRNA experiment, respectively. Grey indicates that selection criteria were not met but the gene was represented in the array. White boxes indicate that the gene was not present in the chip.

**Figure 6:**
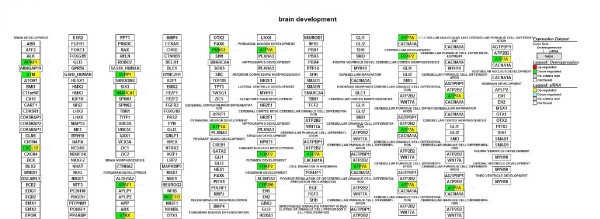
**Gene list involved in the brain development pathway generated by GenMAPP**. The color on the left side of gene box illustrates the gene changes by *ZNF191 *overexpression; the color on the right indicates the gene changes by siRNA-mediated knockdown of *ZNF191*. Red and green indicates up-regulated and down-regulated in *ZNF191 *overexpression experiment, respectively. Yellow and blue indicates up-regulated and down-regulated in siRNA experiment, respectively. Grey indicates that selection criteria were not met but the gene was represented in the array. White boxes indicate that the gene was not present in the chip.

We focused on microarray gene expression data of 1456 genes identified in both transient overexpression and transient knockdown treatment. Using gene-association files from the GO Consortium, MAPPFinder assigns the thousands of genes in the dataset to numerous GO terms. Our analysis was limited to the GO terms that comprise more than eight genes in a class. Several significant (i.e., z score > 2) functional MAPPs were revealed with MAPPFinder and were presented in Table 1. Gene pathways that are involved in the *regulation of kinase activity and angiogenesis *were significantly up-regulated. In contrast, processes associated with *response to DNA damage stimulus and brain development *were down-regulated. Many up-regulated processes are localized to *cellular component *(endoplasmic reticulum) while down-regulated processes belong mainly to the *cellular components *(ribosome, extracellular matrix and cytoplasmic membrane-bound vesicle). Figure 3, 4, 5 and 6 and Table 2 showed the *ZNF191 *overexpression and *ZNF191 *knockdown differentially expressed genes involved in these signaling pathways.

**Table 1:** Identification of significantly changed molecular pathways by mappfinder analysis of the significantly altered genes in both transient overexpression and transient knockdown of the *ZNF191 *gene.

**GOID**	**Pathway Name/GO Term**	**NO. genes**	**% Changed**	**Z-score**
**Up-regulated in overexpression and down-regulated in KD**				
43549	regulation of kinase activity	12	60	2.73
45859	regulation of protein kinase activity	12	60	2.73
165	MAPKKK cascade	8	66.7	2.60
51338	regulation of transferase activity	12	57.1	2.51
65008	regulation of biological quality	15	53.6	2.51
22402	cell cycle process	21	47.7	2.32
1525	angiogenesis	8	61.5	2.31
6952	defense response	13	52	2.19
6508	proteolysis	12	52.2	2.12
48514	blood vessel morphogenesis	8	57.1	2.04
7264	small GTPase mediated signal transduction	10	52.6	1.96
5515	protein binding	108	36.9	2.38
5525	GTP binding	9	60	2.36
30246	carbohydrate binding	10	55.6	2.18
19001	guanyl nucleotide binding	9	56.3	2.11
5783	endoplasmic reticulum	15	48.4	2.01
**Down-regulated in overexpression and up-regulated in KD**				
6066	alcohol metabolic process	14	100	2.56
45449	regulation of transcription	95	77.9	2.45
9719	response to endogenous stimulus	17	94.4	2.40
6974	response to DNA damage stimulus	17	94.4	2.40
19219	regulation of nucleobase\, nucleoside\, nucleotide and nucleic acid metabolic process	95	77.2	2.30
7420	brain development	11	100	2.27
6355	regulation of transcription\, DNA-dependent	89	77.4	2.25
6350	transcription	96	76.8	2.21
3735	structural constituent of ribosome	15	100	2.65
46872	metal ion binding	153	75.7	2.63
43167	ion binding	154	75.1	2.43
43169	cation binding	140	75.3	2.32
3676	nucleic acid binding	129	75.4	2.25
46914	transition metal ion binding	103	76.3	2.17
35091	phosphoinositide binding	10	100	2.16
8270	zinc ion binding	94	76.4	2.09
4930	G-protein coupled receptor activity	9	100	2.05
16023	cytoplasmic membrane-bound vesicle	11	100	2.27
5830	cytosolic ribosome (sensu Eukaryota)	11	100	2.27
5578	proteinaceous extracellular matrix	23	88.5	2.23
31012	extracellular matrix	23	88.5	2.23
5840	ribosome	15	93.8	2.20
5842	cytosolic large ribosomal subunit (sensu Eukaryota)	10	100	2.16
15934	large ribosomal subunit	10	100	2.16
44420	extracellular matrix part	9	100	2.05

**Table 2:** List of genes involved in the signal pathways

				**Fold change**	
				
**Gene symbol**	**ProbeName**	**GenBank**	**Description**	**Overexpression**	**Knockdown**
**Regulation of kinase activity**					
RB1	A_23_P204850	NM_000321	Retinoblastoma 1 (including osteosarcoma)	0.74	1.53
GADD45B	A_23_P142506	NM_015675	Growth arrest and DNA-damage-inducible, beta	1.45	0.68
PKIA	A_23_P31765	NM_006823	Protein kinase (cAMP-dependent, catalytic) inhibitor alpha	0.68	1.41
TIZ/ZNF675	A_24_P931755	D70835	TRAF6-inhibitory zinc finger protein	0.71	1.38
SPRY4	A_24_P269062	NM_030964	Sprouty homolog 4 (Drosophila)	2.12	0.57
DUSP6	A_23_P139704	NM_001946	Dual specificity phosphatase 6	1.56	0.74
RGS4	A_23_P200737	BC000737	Regulator of G-protein signalling 4	1.83	0.65
SPRED2	A_32_P225854	NM_181784	Sprouty-related, EVH1 domain containing 2	1.52	0.72
NRG1	A_23_P315815	NM_004495	Neuregulin 1	1.51	0.74
ZAK/MLTK_HUMAN	A_23_P318300	NM_133646	Sterile alpha motif and leucine zipper containing kinase AZK	0.69	1.56
EDN1	A_23_P214821	NM_001955	Endothelin 1	2.33	0.49
PRKD3	A_24_P165656	NM_005813	Protein kinase D3	0.74	1.56
KITLG	A_24_P133253	NM_000899	KIT ligand	0.68	1.51
C1QTNF6	A_24_P211565	NM_031910	C1q and tumor necrosis factor related protein 6	0.47	1.54
C5	A_23_P71855	NM_001735	Complement component 5	0.42	2.62
MAP4K5	A_23_P205646	BC036013	Mitogen-activated protein kinase kinase kinase kinase 5	0.74	1.39
CCNA1	A_23_P48414	NM_003914	Cyclin A1	2.51	0.54
CDKN2B	A_24_P360674	NM_078487	Cyclin-dependent kinase inhibitor 2B (p15, inhibits CDK4)	1.81	0.71
CKS1B	A_32_P175864	BG205572	CDC28 protein kinase regulatory subunit 1B	1.45	0.64
SERTAD1	A_23_P218463	NM_013376	SERTA domain containing 1	1.51	0.75
**Angiogenesis**					
CEACAM1	A_24_P382319	NM_001712	Carcinoembryonic antigen-related cell adhesion molecule 1 (biliary glycoprotein)	0.60	1.78
COL18A1	A_23_P211212	NM_030582	Collagen, type XVIII, alpha 1	0.74	0.66
CTGF	A_23_P19663	NM_001901	Connective tissue growth factor	3.88	0.24
MYH9	A_23_P57497	BC011915	Myosin, heavy polypeptide 9, non-muscle	1.34	0.75
NRP2	A_24_P50801	AF280546	Neuropilin 2	1.77	0.59
PLXDC1	A_23_P3911	BC036059	Plexin domain containing 1	0.63	1.38
VEGF	A_23_P70398	NM_003376	Vascular endothelial growth factor	0.75	1.31
CXCL12	A_23_P202448	U19495	Chemokine (C-X-C motif) ligand 12 (stromal cell-derived factor 1)	0.73	1.37
CYR61	A_23_P46426	Z97068	Cysteine-rich, angiogenic inducer, 61	1.74	0.44
EDN1	A_23_P214821	NM_001955	Endothelin 1	2.33	0.49
SEMA5A	A_23_P213415	NM_003966	Sema domain, seven thrombospondin repeats (type 1 and type 1-like), transmembrane domain (TM) and sh	0.28	2.26
IL17F	A_23_P167882	NM_052872	Interleukin 17F	1.78	2.01
RUNX1	A_24_P34155	X90980	Runt-related transcription factor 1 (acute myeloid leukemia 1; aml1 oncogene)	1.48	0.74
THBS1	A_23_P206212	X04665	Thrombospondin 1	1.97	0.59
**Brain development**					
APAF1	A_23_P36611	NM_181861	Apoptotic protease activating factor	0.58	1.36
ATM	A_23_P35916	NM_000051	Ataxia telangiectasia mutated (includes complementation groups A, C and D)	0.63	1.35
CXCL12	A_23_P202448	U19495	Chemokine (C-X-C motif) ligand 12 (stromal cell-derived factor 1)	0.73	1.37
SEPP1	A_23_P121926	NM_005410	Selenoprotein P, plasma, 1	0.66	1.36
SMARCA1	A_23_P44244	NM_003069	SWI/SNF related, matrix associated, actin dependent regulator of chromatin, subfamily a, member 1	0.43	1.40
ATRX	A_24_P348660	NM_000489	Alpha thalassemia/mental retardation syndrome X-linked (RAD54 homolog, S. cerevisiae)	0.60	1.49
NOTCH3	A_32_P42895	A_32_P42895	Notch homolog 3 (Drosophila)	0.68	1.46
PRKG1	A_23_P136041	NM_006258	Protein kinase, cGMP-dependent, type I	0.43	1.34
ATP7A	A_23_P217737	NM_000052	ATPase, Cu++ transporting, alpha polypeptide (Menkes syndrome)	0.48	1.52
FOXP2	A_24_P16559	AF467257	forkhead box P2	0.67	1.40
CEP290	A_23_P36865	AB002371	Centrosome protein cep290	0.70	1.43
**Response to DNA damage stimulus**					
ZAK/NP_598407.1	A_23_P318300	NM_133646	Sterile alpha motif and leucine zipper containing kinase AZK	0.69	1.56
BRCA1	A_23_P207400	NM_007294	Breast cancer 1, early onset	0.71	1.3
ATM	A_23_P35916	NM_000051	Ataxia telangiectasia mutated (includes complementation groups A, C and D)	0.63	1.35
ATRX	A_24_P348660	NM_000489	Alpha thalassemia/mental retardation syndrome X-linked (RAD54 homolog, S. cerevisiae)	0.60	1.49
BRCA2	A_23_P99452	NM_000059	Breast cancer 2, early onset	0.62	1.3
BTG2	A_23_P62901	NM_006763	BTG family, member 2	0.72	1.35
Q6P2G2_HUMAN	A_24_P361457	NM_173627	Hypothetical protein FLJ35220 (FLJ35220)	0.63	1.49
RBM14	A_23_P161644	NM_032886	RNA-binding motif protein 14	0.62	1.47
MLH3	A_24_P61520	NM_014381	MutL homolog 3 (E. coli)	0.59	1.45
N4BP2_HUMAN	A_24_P222114	AK001542	Nedd4 binding protein 2	0.52	1.47
PMS1	A_23_P40059	NM_000534	PMS1 postmeiotic segregation increased 1 (S. cerevisiae)	0.65	1.34

### Quantitative Real-Time RT-PCR

**Figure 7:**
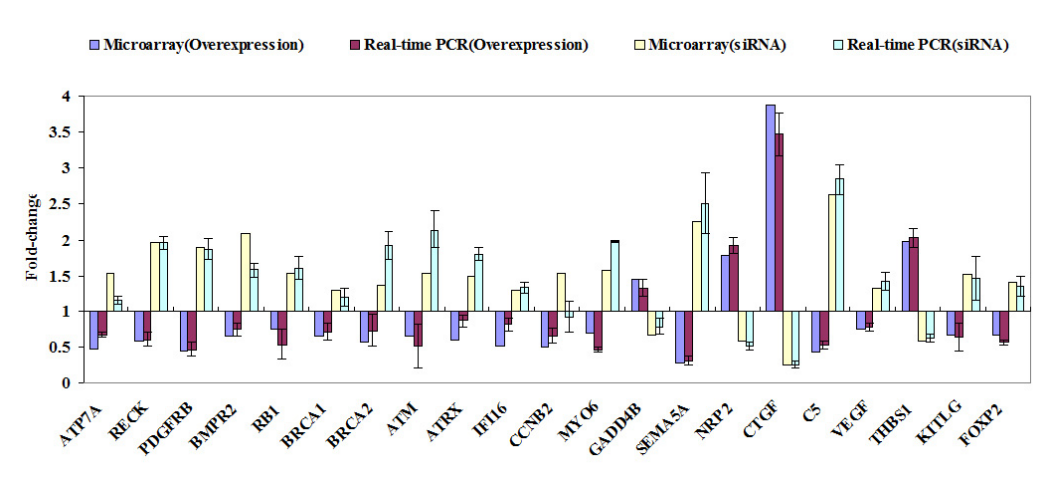
**Quantitative real-time PCR confirmation of the microarray results**. qPCR was performed on 21 genes that showed differential regulation in response to *ZNF191 *overexpression and knockdown by siRNA. Gene expression levels are shown as the mean normalized to the expression of the housekeeping gene *GAPDH*. Each sample was measured in triplicate. *Columns*, mean of three independent experiments; *bars*, SD. Comparison of fold change produced by microarray with relative expression ratio obtained from real-time PCR, with good concordance.

Twenty-one interested genes were selected and subjected to real-time PCR analysis to confirm our microarrays results. As shown in Figure 7, the direction of regulation of *ATP7A, RECK, PDGFRB, BMPR2, RB1, BRCA1, BRCA2, ATM, ATRX, IFI16, CCNB2, MYO6, GADD45B, SEMA5A, NRP2, CTGF, C5, VEGF, THBS1, KITLG and FOXP2 *(expect for *CCNB2*) by the overexpression and knockdown of ZNF191 was consistent with the microarray results.

## Discussion

In this study, we identify genes modified by the ZNF191 transcription factor with a combined strategy of transient overexpression and transient knockdown (KD) in a cellular model (HEK293), using oligonucleotide microarray technology. Several gene pathways were revealed by MAPPfinder to be involved in processes of the regulation of kinase activity, transcription, angiogenesis, brain development and response to DNA damage.

Pathway of regulation of kinase activity was significantly affected (Z-score 2.73). This pathway had a large number of expression changes, mostly due to the regulation of 12 genes (*GADD45B, SPRY4, DUSP6, RGS4, SPRED2, NRG1, EDN1, CCNA1, CDKN2B, CKS1B, SERTAD1 *and *DUSP6*), which were up-regulated in the *ZNF191*-overexpressed cells and down-regulated in the *ZNF191 *knockdown cells. In additional, 8 genes (*KITLG, PKIA, RB1, ZAK, PRKD3, C1QTNF6, C5 *and *MAP4K5*) were down-regulated in the *ZNF191*-overexpressed cells and up-regulated in the *ZNF191 *knockdown cells. A map of the genes involved in regulation of kinase activity was shown in Figure 3. *GADD45B*, originally termed *MyD118*, is first cloned as a myeloid differentiation primary response gene. It can be induced in the absence of protein synthesis following treatment of M1 myeloblastic leukemia cells with differentiation inducers[34], suggesting that GADD45B play a role in hematopoiesis. KITLG is a pleiotropic factor that acts in utero in germ cell and neural cell development, and hematopoiesis[35]. Accordingly, *ZNF191 *has been isolated from bone marrow and promyelocytic leukemia cell lines [26]. These data infer that ZNF191 may play a role in hematopoiesis.

Angiogenesis was another pathway markedly affected by ZNF191 (Z-score 2.31). As shown in Figure 4, *CTGF, CYR61, EDN1, MYH9, NRP2, RUNX1, THBS1 *were up-regulated in the *ZNF191*-overexpressed cells, and down-regulated in the knockdown cells. In addition, *CEACAM1, PLXDC1, CXCL12, SEMA5A *and *VEGF *were down-regulated in the *ZNF191*-overexpressed cells, and up-regulated in the knockdown cells. Angiogenesis, the growth of new blood vessels, is required for a variety of normal proliferative processes. Furthermore, angiogenesis is well established as also playing an important role in neoplastic growth and metastasis. VEGF is a potent stimulator of angiogenesis. *ZNF191 *has been reported to be up-regulated in angiogenic tumor nodules where *VEGF *expression is significantly decreased compared with preangiogenic nodules[36]. In this study, our result in HEK293 cells is consistent with the findings that in human breast carcinoma cells overexpression of *ZNF191 *results in a significant down-regulation of *VEGF*, whereas silencing of *ZNF191 *with small interfering RNA leads to increased *VEGF *expression as well as the same inverse correlation between *ZNF191 *and *VEGF *observed in malignant tissues from human colon and breast biopsies [36]. In addition, thrombospondin-1 (THBS1/TSP-1) has been shown to inhibit angiogenesis through direct effects on endothelial cell migration and survival, and through effects on vascular endothelial cell growth factor bioavailability. Aside from the inhibitory activity of angiogenesis, THBS1 also suppresses tumor growth by activating transforming growth factor beta and affects tumor cell function through interaction with cell surface receptors and regulation of extracellular proteases[37]. The data in this study revealed that overexpression of *ZNF191 *resulted in a significant up-regulation of *THBS1*, whereas silencing of ZNF191 led to decreased *THBS1 *expression in HEK293 cells. Taken together, these data suggest that ZNF191 may participate in negative regulation of angiogenesis. Furthermore, BMPR2 is overexpressed in the majority of human lung carcinomas independent of cell type[38,39]. PDGFR expression in colorectal cancer significantly correlates with lymphatic dissemination[40]. MYO6 (myosin VI) is critical in maintaining the malignant properties of the majority of human prostate cancers diagnosed today[41]. The finding that overexpression of *ZNF191 *significantly down-regulated *BMPR2, PDGFR *and *MYO6*, whereas silencing of ZNF191 increased *BMPR2, PDGFR *and *MYO6 *expression suggests a tumor suppressor role for ZNF191 in human cancers.

The response to DNA damage node was significantly affected (Z-score 2.4), mostly due to the regulation of *BRCA1, BRCA2, ATM, BTG2 *and *ATRX *(Figure 5). The most obvious explanation for this induction was that it was related to initiation of DNA replication. Indeed, DNA repair function is complementary to DNA replication, as the latter process is not error-free and produces mismatches that need to be repaired. However, we reported here that overexpression or elimination of *ZNF191 *affected the upstream regulators of the DNA damage pathway, such as ATM, ATRX, BRCA2 and BTG2. These data suggest that ZNF191 may be involved in the control of DNA damage response in addition to the regulation of DNA replication and concurrent DNA repair. Microarray and real-time PCR showed that RB1, ATM, ATRX and BRCA1 genes were down-regulated in the *ZNF191*-overexpressed cells (Figure 7). Accordingly, RB1 knockdown by siRNA caused the down-regulation of ATM, ATRX and BRCA1[42]. *RB1 *mRNA level was down-regulated in MCF-7 breast carcinoma cells by the overexpression of *ZNF191 *(data not published).

Mice *Zfp191 *transcript is detected early during embryogenesis in ectodermal, endodermal, mesodermal and extra-embryonic tissues. It is particularly observed in the developing central nervous system (CNS)[43]. In rat, *ZNF191 *mRNA is present at high levels in total embryonic head, and abundant in catecholaminergic tissues including substantia nigra, hypothalamus and olfactory bulb as well as peripheral catecholaminergic tissue adrenal medulla[27]. In human, *ZNF191 *transcript is also detected in brain[27]. These studies demonstrate an important role of ZNF191 in brain development. Interestingly, in support with previous studies, our results showed a node affected by *ZNF191 *was the brain development node (Z-score 2.27). 11 genes (*ATP7A, FOXP2, APAF1, ATM, CXCL12, SEPP1, SMARCA1, ATRX, NOTCH3, PRKG1*, and *CEP290*) down-regulated in the *ZNF191*-overexpressed cells and up-regulated in the *ZNF191 *knockdown cells (Figure 6). The human copper-transporting ATP7A is essential for dietary copper uptake, normal development and function of the CNS, and regulation of copper homeostasis in the body[44]. In addition, studies on Alzheimer's disease (AD) suggest an important role for copper in the brain, with some AD therapies focusing on mobilising copper in AD brains[45]. The transport of copper into the brain is complex and involves numerous players, including amyloid precursor protein, A beta peptide and cholesterol[45,46]. Interestingly, in a yeast two-hybrid experiment using the SCAN domain of ZNF191 as bait, we identified that the zinc finger transcription factor ZNF191 can interact with NAE1(NEDD8 activating enzyme E1 subunit 1; amyloid beta precursor protein binding protein 1, 59 kDa) (unpublished data). *FOXP2 *mutations in humans are associated with a disorder that affects both the comprehension of language and its production, speech[47]. *ZNF191 *transcript was abundant in the mesencephalon, a structure that contains the major rat embryonic catecholaminergic tissues[27]. Accordingly, ZNF191 can specifically interact with the widespread TCAT motif which constitutes the HUMTH01 microsatellite in the tyrosine hydroxylase (*TH*) gene (encoding the rate-limiting enzyme in the synthesis of catecholamines)[27]. Allelic variations of HUMTH01 are known to have a quantitative silencing effect on *TH *gene expression and to correlate with quantitative and qualitative changes in the binding by ZNF191[27]. In this study, overexpression of *ZNF191 *resulted in a significant down-regulation of *TH*, whereas knockdown of *ZNF191 *resulted in a non-significant change of *TH *level. The expression level of the TH gene is indistinguishable between the wild-type, *Zfp191*^+/-^, and *Zfp191*^-/- ^mice[32]. These data suggest that other genes may compensate at least in part for the loss of ZNF191 activity, ameliorating the effect of the *ZNF191 *knockdown. Taken together, these data suggest that ZNF191 may be involved in the brain development and the neuropsychiatric diseases.

We have to point out that in the knockdown experiments we can only deduce the level of ZNF191 protein by the level of mRNA of ZNF191 because no antibody against the ZNF191 factor is commercially available and the turn-over of the protein is still unknown. So there is no mean to ascertain whether and when the ZNF191 protein is reduced significantly. In addition, in this study HEK293 cells were used as a cellular model to identify genes modified by the ZNF191 transcription factor. Some other cells could give at least partially over transcriptome modification. Furthermore, the transcriptional response found upon alteration of ZNF191 levels does not necessarily imply that this transcription factor regulates the differentially expressed genes. Many of the observed changes could be downstream and not direct effects of ZNF191 regulation. A proof of direct regulation would require a promoter analysis or some kind of DNA-protein binding experiment or in silico analysis.

## Conclusion

We applied overexpression and siRNA-mediated gene silencing coupled with microarray screening and systematic pathway analysis to obtain insights into the pathways controlled by the target genes. We found that ZNF191 regulated the expression of a large group of genes involved in regulation of kinase activity, angiogenesis, brain development and response to DNA damage. Our analysis reveals that ZNF191 is a pleiotropic factor in hematopoiesis, brain development, and human cancers. Our approach can be used in functional genomics to elucidate the role of other transcription factors.

## Methods

### Cell culture and transfection

HEK-293 cells were cultured in DMEM medium supplemented with 10% fetal bovine serum (FBS) in an atmosphere of 95% air, 5% CO_2 _at 37°C. Cells were plated into 60 mm dishes without antibiotics 24 h prior to transfection. The recombinant plasmids, or control vector, were transfected into HEK-293 cells using Lipofectamine 2000 reagents according to the manufacturer's instructions. HEK-293 cells transfected with each cDNA or siRNA were lysed after 24 h or 48 h to isolate total RNA.

### Plasmids

The cDNA clone of *ZNF191 *was provided by Prof. Long Y. (Genetics Institute, Fudan University, Shanghai, China). *ZNF191 *cDNA was cloned into pcDNA3 (Invitrogen) by using HindIII and XhoI restriction sites. The cloning was verified by DNA sequencing. The hairpin siRNA expression vectors against *ZNF191 *were previously generated [32].

### RNA isolation

Total RNA was extracted using the Trizol reagent (Invitrogen) and purified on RNeasy columns (Qiagen). The amount and quality of RNA preparations were evaluated on the Agilent 2100 Bioanalyzer with RNA6000 Nano Reagents and Supplies (Agilent).

### Microarray hybridization

Five hundred nanograms of RNA were reverse-transcribed to cDNA during which process a T7 sequences was introduced into cDNA. T7 RNA polymerase-driven RNA synthesis was used for the preparation and labeling of cRNA with Cy3 (the reference sample) and Cy5 (the treated sample), respectively. A Qiagen RNeasy Mini Kit (Qiagen Inc., Valencia, CA) was used to purify fluorescent cRNA probes. An equal amount (1 μg) of Cy3 and Cy5 labeled probes were mixed and used for hybridization on one Agilent 4 × 44 K Human Whole-Genome 60-mer oligonucleotide microarrays(Agilent Techonologies Inc., Santa Clara, CA) following the protocol provided by the manufacturer.

### Data acquisition and processing

Microarrays were scanned with a dynamic autofocus microarray scanner (Agilent dual laser DNA microarray scanner, Agilent technologies, Palo Alto, CA, USA), using Agilent-provided parameters (Red and Green PMT were each set at 100%, and scan resolution was set to 10 μm). The Feature Extraction Software v7.5 (Agilent Technologies) was used to extract and analyse the signals. Agilent-provided settings were used except for subtraction of the local background and adjustment of the global background. Poor quality features that were either saturated in the two channels (50% of pixels > saturation threshold) or non-uniform were flagged. Only those features with a signal-to-noise ratio (SNR) of up to 2.6 in at least one channel and significantly different from the local background (two sided Student's t-test < 0.05) were used for further analysis. The mean signal ratio of the two fluorescent intensities (Cy-5 treated cRNA/Cy-3 reference cRNA) is expressed as a logarithm (base 2), providing a relative quantitative gene expression measurement between two samples. For subsequent analysis, we used mean-centered log2 of the normalized (linear & lowess method) sample: reference ratio. Microarray data meeting a criterion (> 1.2-fold change, Flag = 0, S/N > 2.6 and pValueLogRatio < 0.05) were considered to be significant and used for further analysis.

### Pathway analysis

The GeneMapp and MappFinder software packages  were used for pathway analysis of the significantly altered genes identified in both transient overexpression and transient knockdown of the *ZNF191*gene. The MAPPFinder works with GenMAPP and uses the annotations from the Gene Ontology (GO) Consortium to identify global biological trends in gene expression data. MAPPFinder relates microarray data to each term in the GO hierarchy, calculates the percentage of genes changed within each GO term, and generates a gene expression profile at the level of biological processes, cellular components, and molecular functions that allows for identification of specific biological pathways. MAPPFinder then calculates the total number of genes changed within a "parent GO term" and all of its "children" (local MAPPs) using Fisher's exact test. The results are expressed as a "z score" for a particular pathway, and values greater than 2 were considered to be significant. GenMAPP was also used to view and analyze microarray data on biological pathways[33].

### Quantitative real-time PCR

Twenty-one interested genes were selected for validation by real time PCR. cDNAs were prepared from 1 μg of total RNA using AMV reverse transcriptase (Promega) and random primers according to the manufacturer's protocol. For each gene, PCR reactions were run three times on one sample. PCR was performed on Chromo4 Multicolor Real-Time Detection System (Bio-Rad, Inc.) in 20 μl reactions by using the SYBR™ Green PCR Master Mix (TaKaRa). The reaction was repeated for 40 cycles; each cycle consisted of denaturing at 95°C for 15 s, annealing and synthesis at 60°C for 1 min as per manufacturer's instructions. The relative amounts of transcript of the tested genes were normalized by GAPDH endogenous control primers set. The average ratios of relative amounts of transcript in the *ZNF191*-overexpressed cells or *ZNF191 *knockdown cells versus empty vector cells from three replicate runs were calculated. Quantitative values are obtained from the cycle number (Ct value) using Opticon Monitor 3.1 (Bio-Rad) according to the manufacturer's instructions. The threshold cycle (C_T_) values were averaged from the values obtained from each reaction, and each gene was normalized to GAPDH level. Each gene of interest and GAPDH were tested in triplicates to determine the Ct-difference. These Ct values were averaged and the difference between the GAPDH Ct (Avg) and the gene of interest Ct (Avg) was calculated (Ct-diff). The fold change was calculated by the 2-Δ^CT^, where ΔΔCT = (C_T_-treated-C_T_-contrl). C_T_-treated means the signal of an individual gene expressed in *ZNF191*-overexpressed or knockdown samples, while C_T_-control means the signal of the same gene expressed in control samples. The relative expression of the gene of interest was analyzed using the 2-ΔΔ^CT ^method [48]. Because SYBR Green binding is not sequence specific, careful design and validation of each primer pair, as well as cautious manipulation of RNA were undertaken to ensure that only target gene sequence-specific, non-genomic products were amplified by real-time PCR. To achieve this, primers were designed to either span or flank introns. PCR primers are listed in Additional file 4. A dissociation curve analysis was performed at the end of the amplification process in order to verify the specificity of the PCR products. The same PCR products were also evaluated by agarose gel electrophoresis. Data analysis and graphics were the results (mean ± SEM) of three different experiments.

### Semi-quantitative PCR

Semi-quantitative PCRs were performed with Zfp191 specific primers (forward: 5'-ATCAGCGGTGGCCACATCAA-3' and reverse: 5'-GATGGGCCCAACCCAATATAT-3'), which yielded 481 bp products. To serve as an internal control, a 381 bp fragment of the glyceraldehyde-3-phosphate dehydrogenase (GAPDH) cDNA was co-amplified with primers (forward, 5'-AACTTTGGTATCGTGGAAGGA-3'; reverse, 5'-GGAGGAGTGGGTGTCGCTGT-3'). The PCRs were carried out for 25–40 cycles at 94°C for 1 min, 55°C for 1 min, and 72°C for 1 min with a final extension of 5 min at 72°C. All RT-PCR experiments were repeated at least twice and the products were electrophoresed on 1.5% agarose gels containing ethidium bromide.

## Authors' contributions

JL was involved in all experiments, experimental design and bioinformatics analyses. XC and YL co-drafted the manuscript, performed Real RT-PCR. XG and HF assisted in bioinformatics analyses and uploaded data to Gene Expression Omnibus. LQ and ZH co-drafted the manuscript and participated in cell culture. JZ co-drafted the manuscript, made revisions critically for important intellectual content.

## Supplementary Material

Additional file 1 **Regulated genes by ZNF191 overexpression**. Table S1 consists of the regulated probes by *ZNF191 *overexpression, containing the assigned designations (Accession number and sequence name), the fold of changed expression and PValueLogRatio(smaller than 0.05)for these genes. Click here for file

Additional file 2 **Regulated genes by ZNF191 knockdown**. Table S2 consists of the regulated probes by *ZNF191 *knockdown, containing the assigned designations (Accession number and sequence name), the fold of changed expression and PValueLogRatio(smaller than 0.05)for these genes. Click here for file

Additional file 3 **Commonly altered genes by *ZNF191 *overexpression and knockdown**. Table S3 consists of the regulated probes by *ZNF191 *overexpression and knockdown, containing the assigned designations (Accession number and sequence name), the fold of changed expression and PValueLogRatio (smaller than 0.05) for these genes. Click here for file

Additional file 4 **List of primers used for RT-PCR**. Abbreviations: see table 2. Click here for file
