# Supplementary material for: A transcript profiling approach reveals the zinc finger transcription factor ZNF191 is a pleiotropic factor
Source: BMC Genomics. 2009 May 22;10:241. doi: 10.1186/1471-2164-10-241 (PMC2694838; doi:10.1186/1471-2164-10-241)
Supplement: Additional file 4 — List of primers used for RT-PCR. Abbreviations: see table 2. [file 1471-2164-10-241-S4.doc]

**Table S4**: List of primers used for RT-PCR

| **Gene** | **Reference sequence** | **Forward Primer** | **Reverse primer** |
| --- | --- | --- | --- |
| ATP7A | NM_000052 | 5’-tggagtcatcctcttggtcttgt-3’ | 5’-ttagcatgttttctgttcttgttgtaag-3’ |
| ZNF191 | NM_006965.1 | 5’-cattccctaaggcactgtgat-3’ | 5’-ttgaggaacacccatactgaga-3’ |
| RECK | NM_021111 | 5’-gtgttatgcaaaagcaagctgatt-3’ | 5’-cctttcacaaaagtcacagacacaa-3’ |
| PDGFRB | NM_002609 | 5’-gccatataccctaaacttccatcct-3’ | 5’-gtccccagagtgtgatgtgtga-3’ |
| BMPR2 | BC035097 | 5’-ttccggaaaaagaaaggctaga-3’ | 5’-aagagcaaaacacaccaacaaaag-3’ |
| RB1 | NM_000321.2 | 5’-cagaataatcacactgcagcagata-3’ | 5’-cacgcgtagttgaaccttttt-3’ |
| BRCA1 | BC072418.1 | 5’-ttgttgatgtggaggagcaa-3’ | 5’-gattccaggtaaggggttcc-3’ |
| BRCA2 | NM_000059.3 | 5’-agcttactccggccaaaaa-3’ | 5’-ttcctccaatgcttggtaaataa-3’ |
| ATM | NM_000051.3 | 5’-ccaggcaggaatcattcag-3’ | 5’-caatccttttaaatagacggaaagaa-3’ |
| ATRX | NM_000489.3 | 5’-tatgcagagcttgccaaaag-3’ | 5’-aaaatcatctttgtcttcattcagc-3’ |
| IFI16 | NM_005531.2 | 5’-agagccatcttcggactcct-3’ | 5’-tcattttggagattgtgtcttca-3’ |
| CCNB2 | NM_004701.2 | 5’-tggaaaagttggctccaaag-3’ | 5’-tcagaaaaagcttggcagaga-3’ |
| MYO6 | NM_004999.3 | 5’-cttcacccgtacaggtagcc-3’ | 5’-ccacgatctcctgtttccac-3’ |
| GADD45B | NM_015675.2 | 5’-cattgtctcctggtcacgaa-3’ | 5’-taggggacccactggttgt-3’ |
| SEMA5A | NM_003966.2 | 5’-gtgattcatgattgaggaaacg-3’ | 5’-tcctcatgtgtggaaagtgc-3’ |
| NRP2 | NM_201264.1 | 5’-ggacccccaacttggatt-3’ | 5’-atggttaaaaagcgcaggtc-3’ |
| CTGF | NM_001901.2 | 5’-ctcctgcaggctagagaagc-3’ | 5’-gatgcactttttgcccttctt-3’ |
| C5 | NM_001735.2 | 5’-tggctatcagaagagcagagg-3’ | 5’-atggcattgattgtgtcctg-3’ |
| VEGF | NM_001025366.1 | 5’-tgcccgctgctgtctaat-3’ | 5’-tctccgctctgagcaagg-3’ |
| THBS1 | NM_003246.2 | 5’-caatgccacagttcctgatg-3’ | 5’-tggagaccagccatcgtc-3’ |
| KITLG | [NM_000899.3](http://www.ncbi.nlm.nih.gov/entrez/viewer.fcgi?val=NM_000899.3) | 5’-gcgctgcctttccttatg-3’ | 5’-ccttcagttttgacgagagga-3’ |
| FOXP2 | AF467257 | 5’-agctacaagcccttctccaa-3’ | 5’-tgctcttgctgtttcttgtaaaac-3’ |

Abbreviations: see table 3.
